# Commentary: Interpersonal style should be included in taxonomies of behavior change techniques

**DOI:** 10.3389/fpsyg.2016.00894

**Published:** 2016-06-16

**Authors:** Sarah J. Hardcastle

**Affiliations:** Health Psychology and Behavioural Medicine Research Group, Faculty of Health Sciences, School of Psychology and Speech Pathology, Curtin UniversityPerth, WA, Australia

**Keywords:** behavior change techniques, motivational interviewing, health psychology, interpersonal skills, behavior-change interventions

The commentary by Hagger and Hardcastle (2014) contended that current behavior change technique (BCT) taxonomies (e.g., BCTTv1; Michie et al., 2013) have focused almost exclusively on intervention content rather than on the role that interpersonal style plays in promoting behavior change. The commentary demonstrated that interpersonal style is a unique technique and likely interacts with other content-related BCTs in affecting behavior change. However, the previous commentary did not elucidate the relational techniques that could be used alongside content-based techniques to enhance the effectiveness of interventions. The purpose of the current paper is to identify relational techniques that are used in motivational interviewing (MI) (Miller and Rollnick, 2013; Hardcastle et al., 2016) that could be used alongside content-based BCTs to increase the effectiveness of interventions across behavior change interventions regardless of theoretical persuasion.

There have been several previous approaches to examining the components of interventions related to interpersonal style. Core parameters and competencies have been identified as requirements to deliver effective behavior change interventions (Roth and Pilling, 2008; Dixon and Johnston, 2010; Kok et al., 2015). For example, Dixon and Johnston (2010) cite “ability to engage client” and “ability to foster and maintain good intervention alliance” as core competencies. However, these competencies do not identify the particular techniques by which such competencies could be incorporated in interventions. The focus of the current paper is to identify relational techniques to demonstrate such aforementioned competencies.

The relational techniques in MI elucidate its “spirit,” which represents the interpersonal context of the intervention. The “spirit” of MI comprises four key components: collaboration, evocation, radical acceptance and compassion (Miller and Rollnick, 2013). Collaboration refers to a collaborative partnership with clients. Evocation refers to a respectful evocation of a client's own motivation and wisdom. Radical acceptance “honors each person's absolute worth…and supports the person's irrevocable autonomy to choose his or her own way, seeks through accurate empathy to understand other's perspectives and affirms the person's strengths and efforts” (p.19). Compassion is a form of loving that seeks the other's well-being and growth. The underpinning “spirit” of MI and its associated relational techniques could be integrated into interventions regardless of the theoretical affiliation of the intervention. By way of example, we outline five relational techniques that could be effectively integrated into behavior change interventions. A full description of MI relational techniques, including how to provide advice and feedback can be found elsewhere (see Hardcastle et al., 2016).

One of the most commonly used relational techniques used in MI is that of asking *open questions*. Open-ended questions can be used “to engage the client” (e.g., “How can I help with xxx?”), but can also be used to explore past experiences (e.g., “What have you learnt from previous attempts to change?”), explore possible reasons for wanting to change (or not) (e.g., “Why would you want to make this change?”) and as a way of delivering almost any intervention. *Affirmation* is another core relational technique used in MI to acknowledge the client's difficulties, efforts and self-worth (e.g., “Your intention was good even if things didn't turn out as you would like”) which can be effectively used in behavior change interventions regardless of theoretical persuasion. Further, *affirmation* is an effective way to bolster self-efficacy, known to be an important mediator of successful behavior change (McAuley et al., 2003; Bandura, 2004).

The use of *reflective statements* is another commonly used technique in MI whereby the counselor paraphrases client comments by repeating back what the client has said (i.e., “So the message that I'm getting is…”). Reflective statements are powerful as a way to engage the client (i.e., they know they are heard and being listened to) but also in helping the client to think and speak about the behavior change. There are various reflective techniques in MI and it is beyond the remit of this paper to outline them all. However, two specific reflective techniques that may have appeal across interventions, particularly with those less motivated to change behavior (Hardcastle et al., 2015) will be outlined*: double-sided reflection* and *overshooting. Double-sided reflection* captures client ambivalence and communicates to the client that the counselor heard their reasons both for and against change; that the counselor understands the decision is complex (e.g., “On the one hand, you would like to change XX, but on the other hand changing XX would mean giving up Xx”). *Double-sided reflection* could be effectively used alongside the content-based BCT of “pros and cons” to demonstrate empathy and further emphasize the client's decisional balance. The result of using double-sided reflections is that the client feels understood, less resistant and more motivated to change. *Overshooting* is a technique provided by the counselor to argue against change by exaggerating the benefits of or minimizing the harm associated with a risky behavior (e.g., “So you see no benefit in changing XX” or “XX is all positive for you”). Overshooting could be effectively used in interventions that attempt to focus on risks of current behavior. The relational technique of *Overshooting* could be effectively used in conjunction with the following content-based BCTs in the BCTTv1: “Comparative imagining of future outcomes” or “Salience of consequences” to evoke arguments for change and to reduce sustain talk (i.e., the person's own arguments for not changing, for sustaining the status quo (Miller and Rollnick, 2013, p. 7).

We expect these relational techniques to make a contribution to BCT taxonomies and assist in the development of more effective interventions. Descriptions of content-only BCTs, such as goal-setting, do not capture the relational components of the intervention by which that content could be delivered. For example, goal-setting could be delivered empathetically using open-ended questions, affirmation and reflections, or delivered didactically using pencil and paper methods. In summary, BCT taxonomies are generally silent on techniques that relate to the interpersonal style of delivery of interventions and future taxonomies would do well to incorporate these techniques. We anticipate that such relational techniques could have wider appeal and be adopted in a broad spectrum of behavior change interventions alongside other “content” based BCTs.

## Author contributions

SH conceived the ideas presented in the article and drafted the article.

### Conflict of interest statement

The author declares that the research was conducted in the absence of any commercial or financial relationships that could be construed as a potential conflict of interest.
